# Study on the Quality Evaluation of Compound Danshen Preparations Based on the xCELLigence Real-Time Cell-Based Assay and Pharmacodynamic Authentication

**DOI:** 10.3390/molecules23092090

**Published:** 2018-08-21

**Authors:** Guojun Yan, Zhitao Zhu, Liliang Jin, Jun Chen, Hui Xie, Jackelyn Miozzi, Feifei Lei, Xuchao Wei, Jinhuo Pan

**Affiliations:** 1 School of Pharmacy, Nanjing University of Chinese Medicine, Nanjing 210023, China; joun.yan@163.com (G.Y.); zhuzhitao@163.com (Z.Z.); chenjun75@163.com (J.C.); njxh66@163.com (H.X.); FeifeiLei@163.com (F.L.); XuchaoWei@163.com (X.W.); 2 Department of Pathobiological Science, School of Veterinary Medicine, Louisiana State University, Baton Rouge, LA 70803, USA; Liliangjin@163.com; 3 Department of Chemical and Biomolecular Engineering, the Ohio State University, Columbus, OH 43210, USA; Miozzi.5@osu.edu

**Keywords:** compound Danshen preparations, xCELLigence, RTCA (Real-Time Cell-based Assay), quality evaluation

## Abstract

***Objective***: To perform a preliminary study on the quality evaluation of compound Danshen preparations based on the xCELLigence Real-Time Cell-based Assay (RTCA) system and make a pharmacodynamics verification. ***Methods***: The compound Danshen was discussed as a methodological example, and the bioactivity of the compound Danshen preparations were evaluated by real-time cell electronic analysis technology. Meanwhile, an in vivo experiment on an acute blood stasis rat model was performed in order to verify this novel evaluation through the curative effect of dissipating blood stasis. ***Results***: We determined the cell index (CI) and IC50 of the compound Danshen preparations and produced time/dose-dependent cell response profiles (TCRPs). The quality of the three kinds of compound Danshen preparations was evaluated through the RTCA data. The trend of CI and TCRPs reflected the effect of drugs on the cell (promoting or inhibiting), and it was verified that the results correlated with the biological activity of the drugs using a pharmacodynamics experiment. ***Conclusion***: The RTCA system can be used to evaluate the quality of compound Danshen Preparations, and it can provide a new idea and new method for quantitatively characterizing the biological activity of traditional Chinese medicines (TCMs).

## 1. Introduction

The quality control for and evaluation of traditional Chinese medicines (TCMs) have always been a difficult and focal point in the progress of TCMs’ modernization [1]. The current pattern and method of quality control for and evaluation of TCMs are mainly based on the methods and theory of chemical medicines. The concept of “Only Component” for TCMs can hardly control and reflect the safety and efficacy of TCM preparations [2]. It is difficult to guarantee both the intrinsic quality of real stability and controllability and promise a practical clinical application. Biological activity (potency) evaluation is a quality control method that directly inspects the safety and effectiveness of drugs in one step [3]. The adoption of a biological activity evaluation for the quality control of a TCM has become a trend in the field of TCMs [4,5,6].

In this study, a novel bioassay method of Real-Time Cell-based Assay (RTCA) has been adopted. This technology is designed by Acea Biosciences and it is completely different from the traditional cell detection methods that can be achieved without marking and dynamic tracking [7]. RTCA can reflect the strength of a drug’s effect on cells (promoting or inhibiting) by Cell Index values (CI) indirectly and evaluate the biological activity [8]. Currently, many researchers have applied this technology in the field of TCM research [9]. Fu H et al. [10] have shown that RTCA can intuitively reflect the impact of the reactive component acting on the cells in their studies. Our group has applied this technology in blood circulation herbs, such as Panax, Leech, and Safflower, to evaluate their quality [11,12,13]. The results show that the technology can reflect the biological activity of a TCM and is expected to provide a new method for the study of TCM quality standards.

RTCA is a real-time, quantitative resistance test that detects changes in cell morphology and cell differentiation and proliferation and as a microelectronic sensor technology provides dynamic, real-time, label-free cellular analysis for a variety of research applications in drug development, toxicology, cancer, medical microbiology, and virology [9]. This technology allows for increasing productivity and exceeding the limits of endpoint analysis by capturing data throughout the entire time course of an experiment and obtaining more physiologically relevant data. The presence of the cells on top of the electrodes will affect the local ionic environment at the electrode/solution interface leading to an increase in the electrode impedance. The larger the number of cells attached to the electrodes, the larger the increases in electrode impedance. In addition, the impedance depends on the quality of the cells’ interaction with the electrodes [14]. For example, increased cell adhesion or spreading will lead to a bigger change in electrode impedance. Thus, electrode impedance, which is displayed as cell index (CI) values, can be used to monitor cell viability, number, morphology, and adhesion degree in a number of cell-based assays. It is used to indicate a series of physiological states including cell growth, stretching, morphological changes, and the extent of cell death and cell adhesion in a variety of experiments [15]. Then, it is able to evaluate the biological activity of the drug [16].

Compound Danshen preparations include Compound Danshen Tablets (CDTs) and Compound Danshen Dripping Pills (CDDPs). The formula is made from *Radix Salviae Miltiorrhizae*, *Panax Notoginseng*, and *Borneol*. The compound preparation has the effect of promoting blood circulation. It has commonly been used in clinical practice for years as a TCM compound preparation for blood circulation. This research applied the RTCA in the quality evaluation of the compound preparation of Danshen. We chose three kinds of commonly used compound preparations of Danshen as the research objects to evaluate their quality using in vitro biological activity methods. An in vivo experiment on the acute blood stasis rat model was conducted in order to verify the clinical curative effect of dissipating blood stasis. We hope to provide new ideas and new methods that have universality and practicality for quantitatively describing the biological activity of traditional Chinese medicine.

## 2. Results

### 2.1. Screening of Specific Dependent Cell Line

**Figure 1:**
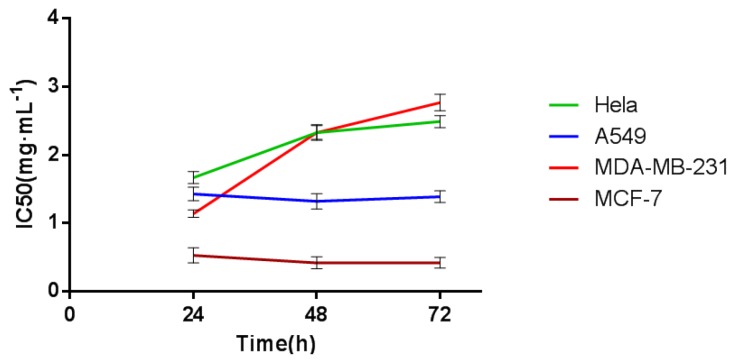
The IC50 values of CDDP on different cell lines at the given time points (24 h, 48 h, 72 h). Data are expressed as x ± s (*n* = 3).

**Figure 2:**
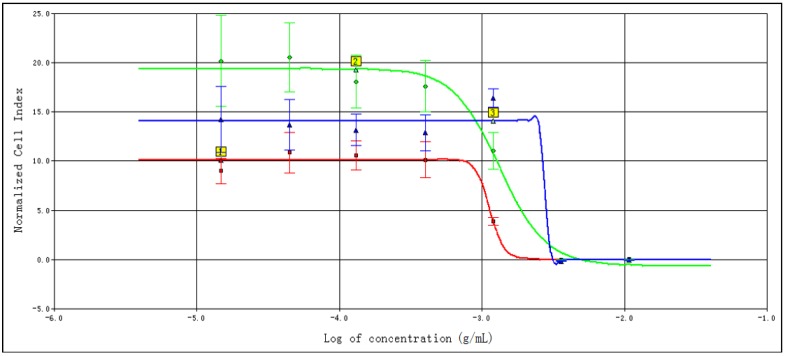
The 50% inhibitory concentration (IC50) calculation curve of MDA-MB-231 cells at the following time points: 24 h (red), 48 h (green), and 72 h (blue).

**Figure 3:**
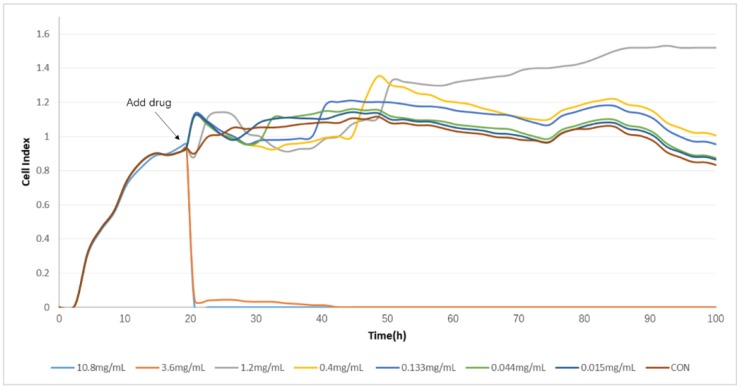
The time/dose-dependent cell response profiles (TCRPs) of the different concentration samples of Compound Danshen Dripping Pills (CDDPs) acting on Hela cells plotted against time with the mean of three experiments. The concentrations are: 10.8 mg/mL (light blue line), 3.60 mg/mL (medium brown line), 1.20 mg/mL (gray line), 0.400 mg/mL (yellow line), 0.133 mg/mL (medium blue line), 0.044 mg/mL (green line), 0.015 (dark blue line), and control (dark brown line).

**Figure 4:**
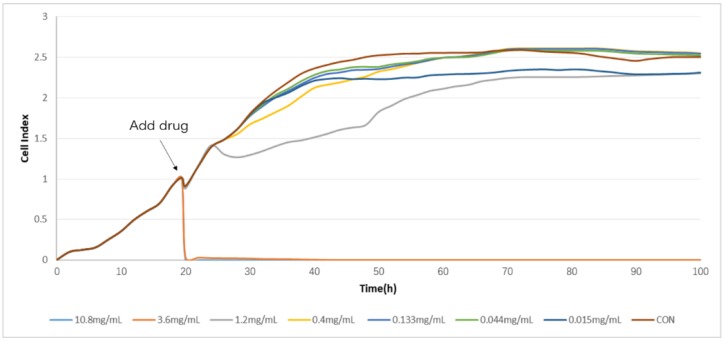
The time/dose-dependent cell response profiles (TCRPs) of the different concentration samples of CDDPs acting on A549 cells plotted against time with the mean of three experiments. The concentrations are: 10.8 mg/mL (light blue line), 3.60 mg/mL (medium brown line), 1.20mg/mL (gray line), 0.400 mg/mL (yellow line), 0.133 mg/mL (medium blue line), 0.044 mg/mL (green line), 0.015 (dark blue line), and control (dark brown line).

**Figure 5:**
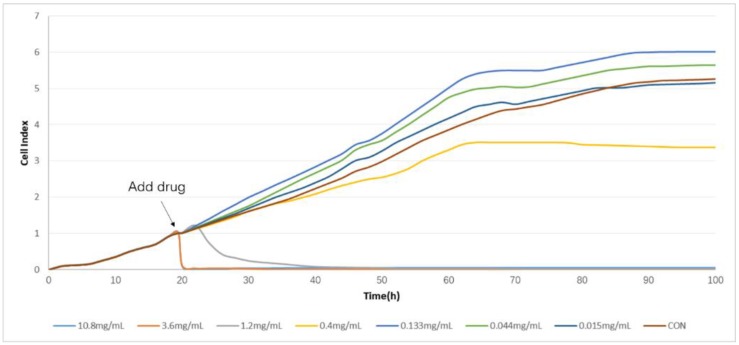
The time/dose-dependent cell response profiles (TCRPs) of the different concentration samples of CDDPs acting on MCF-7 cells plotted against time with the mean of three experiments. The concentrations are: 10.8 mg/mL (light blue line), 3.60 mg/mL (medium brown line), 1.20mg/mL (gray line), 0.400 mg/mL (yellow line), 0.133 mg/mL (medium blue line), 0.044 mg/mL (green line), 0.015 (dark blue line), and control (dark brown line).

**Figure 6:**
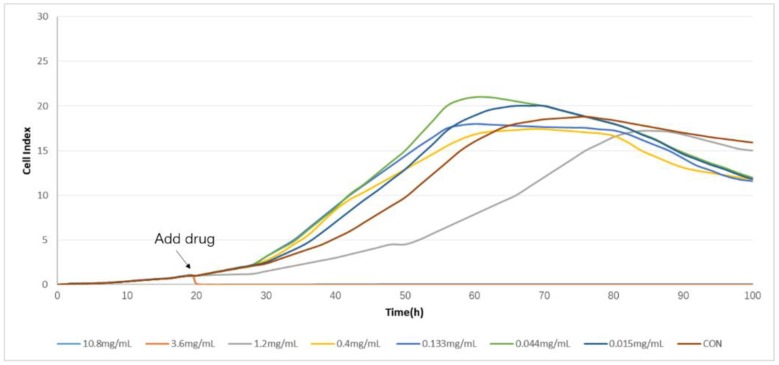
The time/dose-dependent cell response profiles (TCRPs) of the different concentration samples of CDDPs acting on MDA-MB-231 cells plotted against time with the mean of three experiments. The concentrations are: 10.8 mg/mL (light blue line), 3.60 mg/mL (medium brown line), 1.20mg/mL (gray line), 0.400 mg/mL (yellow line), 0.133 mg/mL (medium blue line), 0.044 mg/mL (green line), 0.015 (dark blue line), and control (dark brown line).

In this experiment, CDDP and CDT were used in clinical research as blood circulation stasis TCM compound preparations. The corresponding clinical indications were the same, so the selected drugs of the screening cells are CDDPs. It indicated that the time/dose-dependent cell response profiles (TCRPs) of the same sample acting on different cell lines were quite different with RTCA. The 50% inhibitory concentration (IC50) was calculated by the RTCA software. The value is different at different time points; it indicates that the cellular effects are inconsistent with the time changes. After some time point, the effect will be relatively stable [9]. The results are shown in Figure 1 and Figure 2. According to the TCRPs results, CDDPs significantly inhibited the proliferation of these four kinds of cells. However, there were some differences in the cells’ sensitivity and regularity to CDDPs (Figure 3, Figure 4, Figure 5 and Figure 6). The sensitivity and regularity of MCF-7 and A549 cells was better compared with those of MDA-MB-231 and Hela cells. MCF-7 was much more sensitive than A549. Through analysis and comparison, lung cancer A549 cells were selected as the subjects and used for the following measure of compound Danshen preparations.

### 2.2. Determination of TCRPs of Compound Danshen Preparations

**Figure 7:**
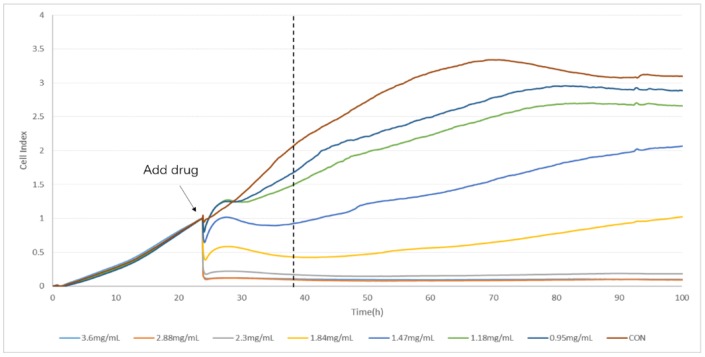
The time/dose-dependent cell response profiles (TCRPs) of the different concentration samples of CDDPs acting on A549 cells plotted against time with the mean of three experiments. The concentrations are: 3.60 mg/mL (light blue line), 2.88 mg/mL (medium brown line), 2.30 mg/mL (gray line), 1.84 mg/mL (yellow line), 1.47 mg/mL (medium blue line), 1.18 mg/mL (green line), 0.95 (dark blue line), and control (dark brown line).

**Figure 8:**
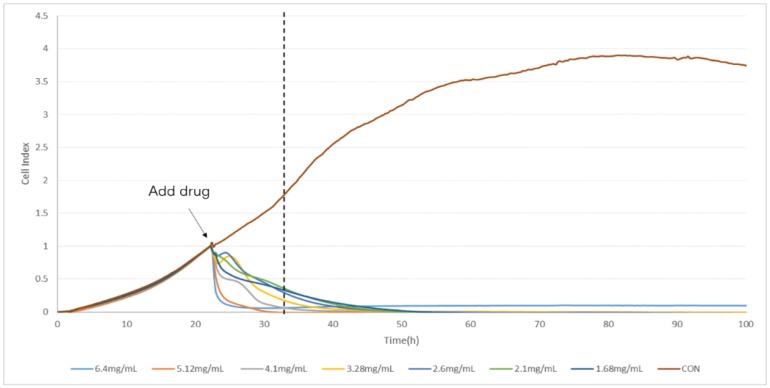
The time/dose-dependent cell response profiles (TCRPs) of the different concentration samples of Anhui Huatuo State pharmaceutical Compound Danshen Tablets (AHSP-CDTs) acting on A549 cells plotted against time with the mean of three experiments. The concentrations are: 6.40 mg/mL (light blue line), 5.12 mg/mL (medium brown line), 4.10 mg/mL (gray line), 3.28 mg/mL (yellow line), 2.60 mg/mL (medium blue line), 2.10 mg/mL (green line), 1.68 (dark blue line), and control (dark brown line).

**Figure 9:**
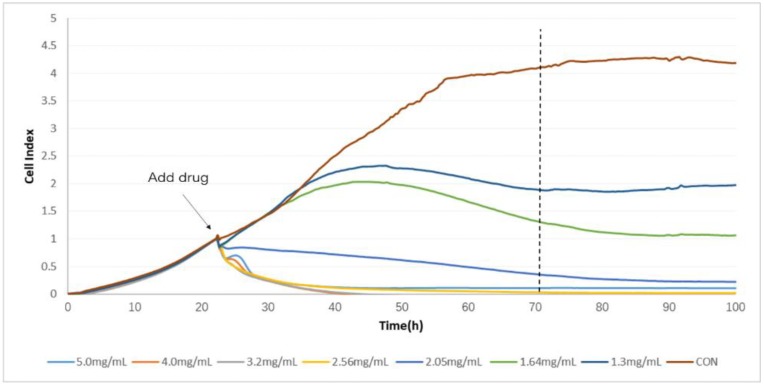
The time/dose-dependent cell response profiles (TCRPs) of the different concentration samples Shanghai Yellow Sea pharmaceutical Compound Danshen Tablets (SYSP-CDT) acting on A549 cells plotted against time with the mean of three experiments. The concentrations are: 5.00 mg/mL (light blue line), 4.00 mg/mL (medium brown line), 3.20 mg/mL (gray line), 2.56 mg/mL (yellow line), 2.05 mg/mL (medium blue line), 1.64 mg/mL (green line), 1.30 (dark blue line), and control (dark brown line).

**Figure 10:**
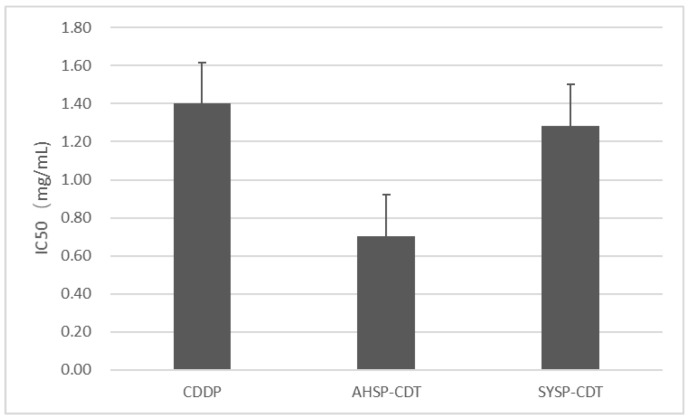
The IC50 values of the three compound Danshen preparations on A549 cells at the given time points marked with dashed lines in Figure 7, Figure 8 and Figure 9. Data are expressed as x ± s (*n* = 3).

The TCRPs curves of compound Danshen preparations acting on the A549 lung cancer cell line, shown in Figure 7, Figure 8 and Figure 9, shows the cells’ dynamic growth trend in real time. Compared with the blank, compound Danshen preparations showed significant inhibition of the growth of A549 lung cancer cell lines, which indirectly reflects the drug’s promoting or inhibiting cell growth by the CI. With the increase of CI, more and more cells adhered to the surface of the electrode, and the inhibition effect of the compound Danshen preparations on cell line growth weakened, meaning that the biological activity of the drug had become lower. As seen in Figure 1, Figure 2, Figure 3 and Figure 4, cells have nearly died at a CDDP concentration of 2.3 mg/mL, a dry extract concentration of CDT from Anhui Huatuo State pharmaceutical company (Bozhou, China) (AHSP-CDT) of 1.68 mg/mL, and a dry extract concentration of CDT from Shanghai Yellow Sea pharmaceutical Co., Ltd. (Shanghai, China) (SYSP-CDT) at 2.56 mg/mL. Dry extract concentrations of the two Compound Danshen Tablets were based on a conversion of the results of a clinically equivalent dose of CDDPs. The IC50 values of three kinds of compound Danshen preparations were calculated and are shown in Figure 10. The strongest biological activity of the three preparations is AHSP-CDT followed by SYSP-CDT and CDDP.

### 2.3. Results of Pharmacodynamic Authentication

#### 2.3.1. Effect of Danshen Preparations on Whole Blood Viscosity (WBV) and Plasma Viscosity (PV) of Acute Blood Stasis Rats

**Figure 11:**
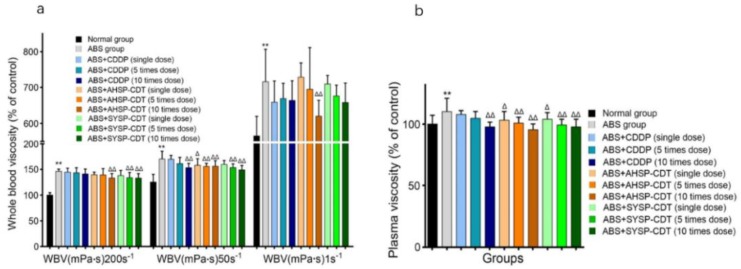
Bar chart of Danshen preparations’ effect on whole blood viscosity (WBV) (**a**) and plasma viscosity (PV) (**b**) of acute blood stasis rats. Compared with the normal group, * *p* < 0.05, ** *p* < 0.01; Compared with the acute blood stasis (ABS) group, Δ *p* < 0.05, ΔΔ *p* < 0.01.

The acute blood stasis (ABS) model increases the WBV and PV readouts and any effective treatment will reduce these values ideally down to the baseline reading of the untreated normal group. Compared with the normal group, the WBV and PV of the ABS group were significantly increased, and the difference was statistically significant (*p* < 0.01). Compared with the ABS group, the WBV and PV of the administration group have a certain degree of reduction. In the WBV, the 200 s^−1^ difference of AHSP-CDT is 10 times that of the dose group and that of SYSP-CDT is 5 times that of the dose group. The 10 times the dose group difference is statistically significant (*p* < 0.01). The 50 s^−1^ difference of the CDDP group is 10 times that of the dose group. The AHSP-CDT single-dose group, 5-fold dose group, and 10-fold dose group and the SYSP-CDT 5-fold dose group and 10-fold dose group differences were statistically significant (*p* < 0.05), and the 1 s^−1^ difference of the AHSP-CDT 10-fold dose group was statistically significant (*p* < 0.01). In PV, in addition to the CDDP single-dose group and the 10 times the dose group, the differences of the other group were statistically significant (*p* < 0.05) (Figure 11).

#### 2.3.2. Effect of Danshen Preparations on the Erythrocyte Sedimentation Rate (ESR), Hematocrit (HCT), Erythrocyte Aggregation Index (EAI), and Carson Viscosity of Acute Blood Stasis Rats

**Figure 12:**
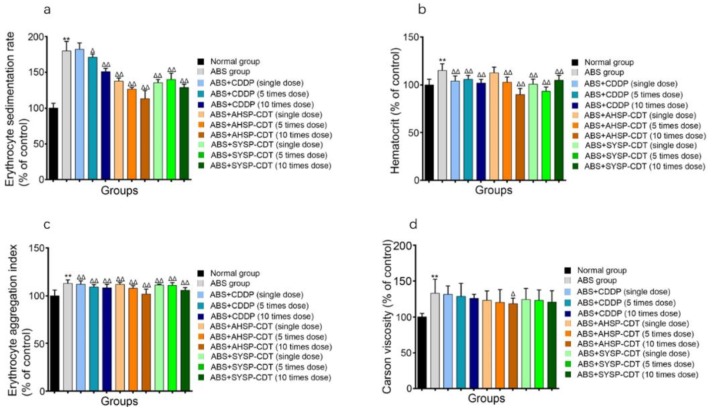
Bar chart of Danshen preparations’ effect on the erythrocyte sedimentation rate (ESR) (**a**), hematocrit (HCT) (**b**), erythrocyte aggregation index (EAI) (**c**) and Carson viscosity (**d**) of acute blood stasis rats. Compared with the normal group, * *p* < 0.05, ** *p* < 0.01; Compared with the ABS group, Δ *p* < 0.05, ΔΔ *p* < 0.01.

The ABS model increases the ESR, HCT, EAI, and Carson viscosity readouts and any effective treatment will reduce these values. Compared with the normal group, the ESR, HCT, EAI, and Carson viscosity of the ABS group have increased to some extent with statistical significance (*p* <0.01). Compared to the ABS group, the ESR, HCT, EAI, and Carson viscosity of each administration group have a certain degree of decrease. In the ESR, in addition to the CDDP single-dose group, the other administration groups are statistically significant (*p* < 0.05). In HCT, in addition to the AHSP-CDT single-dose group, the other administration groups were statistically significant (*p* < 0.01). In the EAI, each administration group was statistically significant (*p* < 0.01). In Carson viscosity, the AHSP-CDT 10 times the dose group was statistically significant (*p* < 0.05) (Figure 12).

#### 2.3.3. Effect of Danshen Preparations on the Prothrombin Time (PT), Thrombin Time (TT), Activated Partial Thromboplastin Time (APTT), and Fibrinogen (FIB) of Acute Blood Stasis Rats

**Figure 13:**
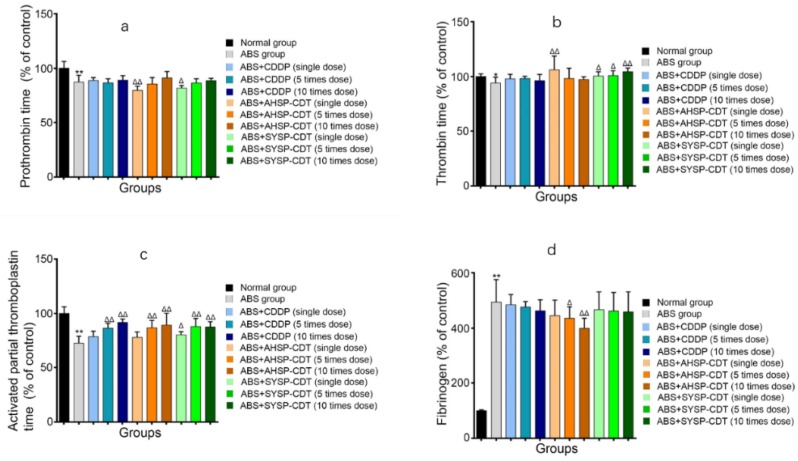
Bar chart of Danshen preparations’ effect on the prothrombin time (PT) (**a**), thrombin time (TT) (**b**), activated partial thromboplastin time (APTT) (**c**), and fibrinogen (FIB) (**d**) of acute blood stasis rats. Compared with the normal group, * *p* < 0.05, ** *p* < 0.01; Compared with the ABS group, Δ *p* < 0.05, ΔΔ *p* < 0.01.

The ABS model reduces the PT, TT, and APTT readouts and increases the FIB readout; any effective treatment will increase the PT, TT, and APTT values and reduce the FIB value. Compared with the normal group, the PT, TT, and APTT within the ABS model group was shortened, and the difference was statistically significant (*p* < 0.05); FIB significantly increased, and the difference was significant (*p* < 0.01). Compared with the ABS group, the PT, TT, and APTT in each administration group was extended to some extent. In PT, the differences of the AHSP-CDT single-dose group and the SYSP-CDT single-fold dose group were statistically significant. In the TT, differences of the AHSP-CDT single-dose group and the SYSP-CDT single dose group, 5 times dose group, and 10 times dose group were statistically significant. In APTT, in addition to the CDDP single-dose group and the AHSP-CDT single-dose group, the differences of other groups were statistically significant. In FIB, the differences of the AHSP-CDT 5 times the dose group and 10 times the dose group were statistically significant (Figure 13).

#### 2.3.4. The Total Value of the Blood Circulation Promotion Effect of Danshen Preparations Based on Multi-Index Comprehensive Index Value.

**Figure 14:**
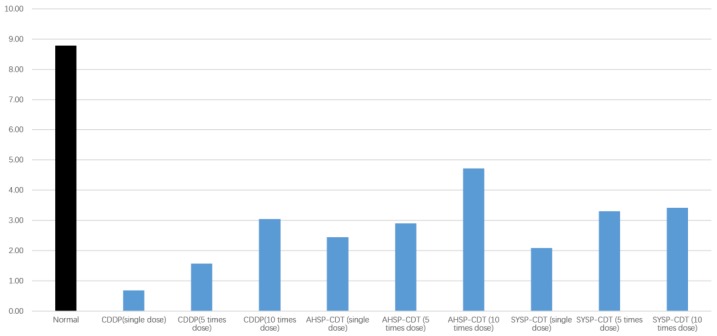
The total value of the blood circulation promotion effect of compound Danshen preparations based on multi-index comprehensive index value.

Based on the multi-index comprehensive index value, which comprises the single-process multiple indicators WBV, PV, ESR, HCT, EAI, Carson viscosity, TT, PT, APTT, and FIB, the total value of each administration group’s blood circulation promotion effect was calculated. The results are shown in Table 1 and Figure 14. The blood circulation promotion effect order of the administration groups is: AHSP-CDT (10 times dose) > SYSP-CDT (10 times dose) > SYSP-CDT (5 times dose) > CDDP (10 times dose) > AHSP-CDT (5 times dose) > AHSP-CDT (single dose) > SYSP-CDT (single dose) > CDDP (5 times dose) > CDDP (single dose). As can be seen from the results, the blood circulation promotion effect of AHSP-CDT is superior to that of SYSP-CDT, and the blood circulation promotion effect of CDDPs is inferior to the two different types of tablets mentioned above.

## 3. Discussion

The quality evaluation of traditional Chinese medicines (TCMs) is difficult due to their complicated effective components; some of them are specific and some of them are undefined [1,2,17,18]. A determination of several ingredients in a TCM cannot represent the whole preparation and reflect the preparation’s holistic effect. Therefore, we want to perform a tentative study through cell effect detection to make a quality evaluation for TCMs. An RTCA provides a dynamic, real-time, label-free cellular analysis for a variety of research applications in drug development. The holistic effect of compound Chinese medicines can be quantitatively detected through an RTCA. We took compound Danshen preparations as representative preparations to build a novel method for quality evaluation of compound Chinese medicines. We hope to provide new ideas and new methods that have universality and practicality for quality evaluation, which reflect the biological activity of instead of the chemical components in traditional Chinese medicines.

Chinese compound medicines’ effect on cells is multi-influenced. Different cell lines grow to different densities under the effect of medicines. These differing growth characteristics indicate that the same compound Danshen preparation has a different effect on different cell lines. The TCRPs of some cells may have no regularity with the concentrations, and they might even show a biphasic response, such as cell death at high concentrations and cell growth at lower concentrations (Figure 1). This may correlate to cell adherence, which mainly influences the detection of the CI value [7]. Therefore, a cell line screening before a quality evaluation of a Chinese compound medicine is necessary. An appropriate sensitivity and a clear regularity of the cell effect is critical. The mechanism of action for cell lines by Chinese compound medicines is complicated due to their complicated components. There seem to be some short term effects on the cell lines, which may indicate that this effect results from the accumulation of compound ingredients in the mitochondria [19,20]. Deep research with more cell models is needed, and more research on the mechanisms underlying the effects of Chinese compound medicines on cells using xCELLigence should be conducted [7,8,21,22].

The objective of this study was to evaluate the quality of compound Danshen preparations by means of real-time cell electronic analysis and perform a verification with in vivo pharmacodynamics. The effect of compound Danshen preparations on cancer cell lines was related to the pharmacological action of blood activation and stasis resolution. A number of references [23,24] show that Chinese medicines with efficacy in blood activation and stasis resolution can improve microcirculation, increasing vascular permeability and inhibiting tumor angiogenesis. The detected CI values on A549 cells by an RTCA show the inhibition effect of compound Danshen preparations on cancer cell lines. The cell and rat experiment results also show that the pharmacodynamics effect on the acute blood stasis model increased as the inhibition effect on A549 cells was enhanced. Therefore, the CI values reflecting the Chinese compound preparation’s holistic effect could be used to quantitatively evaluate the quality of Chinese medicine.

Through the theoretical analysis and documentary accumulation, we demonstrate that real-time cellular electronic analysis techniques can be used in the field of traditional Chinese medicine analysis as quality control methods for investigating drug safety and efficacy. Put the detection plate into the real-time cell electronic analyzer, set the parameters, measure the TCRPs after each solution has acted on the cell line with real-time cell analysis techniques, and calculate the IC50 at the same time. Select the cell lines better depending on the compound Danshen preparations. The rats were subjected to an acute blood stasis model and verified by pharmacodynamics. Through repeated experiments, we found that the results show a real-time cell electron analysis and in vivo pharmacodynamics can quantitatively characterize the biological activity of traditional Chinese medicines.

## 4. Materials and Methods

### 4.1. Materials

CDDPs (25 mg per pill) were purchased from Tianjin Tasly Pharmaceutical Group Limited by Share Ltd. and prescribed as 10 pills three times a day. Compound Danshen Tablets (0.25 g per piece) were purchased from Anhui Huatuo State pharmaceutical company and prescribed as three pieces three times a day. Compound Danshen Tablets (0.45 g per piece) were purchased from Shanghai Yellow Sea pharmaceutical company limited. The preparation above was identified to comply with the provisions of Chinese Pharmacopoeia (2015). Human lung cancer cells (A549), human cervical cancer cells (Hela), human breast cancer cells (MCF-7), and human breast cancer cells (MDA-MB-231) were purchased from American Type Culture Collection (ATCC). Cells were cultured in a humidified incubator at 37 °C with 5% CO_2_ inside according to the optimal media and growth conditions specified by ATCC [25]. Sprague Dawley (SD) rats, 110, female, body weight (200 ± 20) g. The Activated partial thromboplastin time (APTT) assay kit, Prothrombin time (PT) assay kit, Thrombin (TT) kit, and Fibrinogen (FIB) assay kit were all purchased from Nanjing Jiancheng Institute of Biotechnology.

### 4.2. Preparation of Samples

Preparation of the solution of Compound Danshen Dripping Pills (CDDPs): Accurately weighed CDDP at 1.35 g, placed in a 25 mL volumetric flask, added an appropriate amount of purified water, processed it ultrasonically for 30 min to dissolve, then added clean water to the mark. The solution was stored at 4 °C as a storage backup.

Preparation of the solution of AHSP-CDT: Accurately weighed 7.5 g of CDT (Anhui Huatuo State pharmaceutical Company), added 100 mL of 60% ethanol, extracted for 1 h, filtered, obtained the filtrate, placed the filtrate in an evaporating dish, and evaporated it to yield a 4.9191-g sample.

Preparation of the solution of SYSP-CDT: Accurately weighed 13.5 g of CDT (Shanghai Yellow Sea pharmaceutical company limited), added 100 mL of 60% ethanol, extracted for 1 h, filtered, obtained the filtrate, placed the filtrate in an evaporating dish, and evaporated it to yield a 3.7249-g sample.

### 4.3. Cell Line Screening

An RTCA detected the time/dose-dependent cell response profiles (TCRPs) of the samples. Fifty microliters (50 µL) of growth media were added into each well of an E-plate of 96 wells with a pipette (cell culture plate with electronic chips). One hundred milliliters (100 mL) of specific cell suspension was added to each well at a concentration of 2 × 10^5^ cells/mL. The detection plate with a number of cells was let to stand at room temperature for about 30 min, then the cells were incubated. Continue to read until the cells present a stable baseline (about 18–24 h).

In addition to the blank control group, the solution of Compound Danshen Dripping Pills (CDDP) was added to the wells of the rest of the treatment group so that the liquid’s final concentrations inside the wells were separately 0.015, 0.044, 0.133, 0.4, 1.2, 3.6, and 10.8 mg/mL. The detection plate was put into the real-time cell electronic analyzer, the parameters were set, the TCRPs were measured after each solution had acted on the cell line with real-time cell analysis techniques, and the IC50 values were calculated at the same time. Better cell lines were selected depending on the compound Danshen preparations.

### 4.4. Determination of TCRPs of Compound Danshen Preparations

The method is the same as for the specific dependent cell screening. Each pill of Compound Danshen Dripping Pills weighs 27 mg, and was prescribed as 10 pills three times a day. Each tablet of AHSP-CDT weighs 0.25 g, and was prescribed as three pills three times a day. Each tablet of SYSP-CDT weighs 0.45 g, and was prescribed as three pills three times a day. TCRPs were determined with different concentrations of 0.95, 1.18, 1.47, 1.84, 2.3, 2.88, and 3.6 mg/mL when adding the solution of Compound Danshen Dripping Pills (CDDP) to the wells of the cell culture plate. TCRPs were determined for Compound Danshen Tablets’ dry extract concentrations (Anhui Huatuo State pharmaceutical companies), which were separately 1.68, 2.1, 2.6, 3.28, 4.1, 5.12, and 6.4 mg/mL when added to the cell culture plate with the clinical equivalent dose conversion. TCRPs were determined for Compound Danshen Tablets’ dry extract concentrations (Shanghai Yellow Sea pharmaceutical Co., Ltd., Shanghai, China) of 1.3, 1.64, 2.05, 2.56, 3.2, 4.0, and 5.0 mg/mL separately.

## 5. Pharmacodynamics Studies In Vivo of Compound Danshen Preparation

### 5.1. Animal

Sprague Dawley (SD) rats, 110, female, body weight (200 ± 20) g were provided by the Experimental Animal Center of Zhejiang Province (SPF grade), license number SCXK (Zhejiang) 2014-0001.

### 5.2. Reagent

Adrenaline hydrochloride injection (Shanghai Hefeng Pharmaceutical Co., Ltd., Shanghai, China), batch number: W140201; chloral hydrate (Sinopharm Chemical Reagent Co., Shanghai, China), batch number 20100709; sodium citrate (Nanjing Chemical Reagent Co., Ltd., Nanjing, China), batch number: 050 580 052; activated partial thromboplastin time (APTT) determination kit, Nanjing Jiancheng Institute of biotechnology, batch number: 20140715, prothrombin time (PT) determination kit, Nanjing Jiancheng Institute of biotechnology, batch number: 20140715; thrombin (TT) kit Nanjing Jiancheng Institute of Biotechnology, batch number: 20140715; fibrinogen (FIB) determination kit, Nanjing Jiancheng Institute of Biotechnology, batch number: 20140718; CDDP (Tianjin Tasly Pharmaceutical Group Co., Ltd., Tianjin, China), batch number: 130 420; Compound Danshen Tablets, Anhui Huatuo State pharmaceutical companies, batch number: 20140313; Compound Danshen Tablets (Shanghai Yellow Sea pharmaceutical Co., Ltd., Shanghai, China), batch number: 140,117. CDDPs were dissolved with a moderate amount of purified water. Compound Danshen Tablets were extracted with 60% ethanol and filtered. Ethanol was put into the subsequent filtrate evaporates and diluted to a certain volume.

### 5.3. Preparation of Acute Blood Stasis Model

Reference [26,27]. Rats were given an adrenaline hydrochloride injection twice, 0.8 mg/kg, at intervals of 4 h. After 2 h of the first subcutaneous administration of adrenaline hydrochloride, the rats were placed in 0–2 °C ice water and swam for 4 min, leading to the acute blood stasis model. After the last subcutaneous injection of adrenaline hydrochloride, feed water was added to fasted rats. Sixteen to 18 h later, measurements of related indicators of blood rheology and coagulation were made by the abdominal aortic method.

### 5.4. Animal Grouping and Administration

The rats were randomly divided into 11 groups, and every group contained 10 rats. Group 1 was the normal group and group 2 was the untreated acute blood stasis (ABS) group. Groups 3 to 5 for the CDDP were the high-, medium-, and low-dose groups. Groups 6 to 8 were the Compound Danshen Tablets from Anhui Huatuo State pharmaceutical companies (AHSP-CDT) high-, medium-, and low-dose groups. Groups 9 to 11 were the Compound Danshen tablets from Shanghai Yellow Sea pharmaceutical Co., Ltd. (SYSP-CDT) high-, medium-, and low-dose groups. The CDDP high-, medium-, and low-dose groups’ dose was, respectively, 10 times the amount of the clinical equivalent dose (0.8 g/kg), 5 times the amount (0.4 g/kg), and a single amount (0.08 g/kg). The compound Danshen tablets (Anhui Hua State pharmaceutical companies) high-, medium-, and low-dose groups’ dose in terms of the amount of the original formulation was 10 times the amount of clinical equivalent dose (2 g/kg), 5 times the amount (1 g/kg), and a single amount (0.2 g/kg). The SYSP-CDT high-, medium-, and low-dose groups’ dose in terms of the amount of the original formulation was 10 times the amount of clinical equivalent dose (3.6 g/kg), 5 times the amount (1.8 g/kg), and a single amount (0.36 g/kg). The normal group and the untreated ABS group were given an equal volume of distilled water by lavage administration every morning and evening for 7 days. On the third day, all groups except the normal group copied the acute blood stasis model according to the corresponding modeling method. After 30 min of the seventh administration, related indicators of blood rheology and coagulation were measured by the abdominal aortic method [28].

### 5.5. Measuring Indicators of Blood Rheology and Coagulation

Rats were exposed to anesthesia at 10% chloral hydrate and blood was taken from the abdominal aorta with sodium citrate (3.8%) 1:9 anticoagulation. One milliliter (1 mL) of whole blood was drawn into a hematocrit tube for 1 h and the erythrocyte sedimentation rate (ESR) was read as well as recorded. The whole blood was centrifuged at a rate of 3000 r/min for 30 min, and the hematocrit (HCT) was read and recorded. Next, an SA-5000 automatic blood rheology tester was used to measure the whole blood viscosity (WBV) and the erythrocyte aggregation index (EAI). The whole blood was centrifuged at a rate of 3000 r/min for 10 min, blood plasma was drawn, and the SA-5000 automatic blood rheology tester was used to measure the plasma viscosity (PV). Relevant indicators were measured by using an LG-PABER-1 with a platelet aggregation coagulation factor analyzer. The specific operation sequences follow the instructions for APTT, TT, PT, FIB.

### 5.6. Statistical Method

The data were processed by the SPSS16.0 software. Experimental results were represented as x ± s and compared with model groups which used Dennett’s test of ANOVA comparison that indicated a statistically significant difference at *p* < 0 05.

In this study, there are a lot of indicators that needed to be measured, which is why we use an integrated multi-index comprehensive index method to value the blood circulation effect. A single process for indicators such as ESR, HCT, WBV, PV, EAI, APTT, PT, TT, and FIB calculates the total value of the blood circulation promotion effect of each administration group. First, the index was standardized, when the index value (Value) falls after modeling, and the standardized value of each index V standardized = (V administration − V model)/V model. When the index value is higher than that of the normal group after modeling, V standardized = (V model − V administration)/V models. The weight of standardized indicators is referred to as the expert scoring method, and it independently gives a weighting factor for each indicator. Consulting more than 50 articles related to acute blood stasis from nearly the last 10 years [28], 48 detected WBV, 43 detected PV, 46 detected HCT, 25 detected the FIB, 13 detected ESR, 12 detected PT, 11 detected APTT, 5 detected TT, 3 detected EAI, and 3 detected Carson viscosity. Combining the expert scoring method with the frequency of each indicator, which was detected from related acute blood stasis references in the past 10 years, this study takes the weight coefficients. WBV, PV, and HCT take a weight of 4, FIB takes a weight of 3, ESR, PT, and APTT take a weight of 2, and TT, EAI, and Carson viscosity take a weight of 1. The total value of the blood circulation promotion effect = Σ standardized value × weight coefficient.

## Figures and Tables

**Table 1:** The total value of the blood circulation promotion effect of Danshen preparations based on multi-index comprehensive index value.

Group	Vs a					Vs b	Vs c			Vs d			Total Value of Blood Circulation Promotion Effect (4a + 3b + 2c + 1d)
	WBV200	WBV50	WBV1	PV	HCT	FIB	ESR	PT	APTT	TT	EAI	Carson Viscosity	
Normal	0.32	0.26	0.21	0.09	0.13	0.80	0.44	0.14	0.38	0.06	0.11	0.25	8.78
CDDP (single dose)	0.01	0.00	0.08	0.02	−0.01	0.02	−0.01	0.01	0.08	0.04	0.01	0.01	0.68
CDDP (5 times dose)	0.02	0.05	0.06	0.05	0.05	0.03	0.05	−0.01	0.19	0.04	0.03	0.03	1.57
CDDP (10 times dose)	0.03	0.10	0.07	0.11	0.16	0.06	0.16	0.02	0.26	0.02	0.04	0.05	3.05
AHSP-CDT (single dose)	0.04	0.07	−0.02	0.06	0.23	0.10	0.23	−0.09	0.07	0.13	0.01	0.07	2.45
AHSP-CDT (5 times dose)	0.05	0.08	0.03	0.08	0.11	0.12	0.30	−0.02	0.20	0.05	0.04	0.09	2.9
AHSP-CDT (10 times dose)	0.09	0.08	0.13	0.13	0.22	0.20	0.37	0.04	0.23	0.03	0.10	0.11	4.72
SYSP-CDT (single dose)	0.05	0.06	0.01	0.06	0.12	0.05	0.25	−0.06	0.11	0.07	0.01	0.06	2.09
SYSP-CDT (5 times dose)	0.08	0.10	0.06	0.10	0.19	0.06	0.22	−0.01	0.21	0.07	0.02	0.07	3.3
SYSP-CDT (10 times dose)	0.09	0.12	0.08	0.11	0.09	0.07	0.28	0.01	0.20	0.11	0.06	0.09	3.41
